# Supplementary material for: A novel fragile X syndrome mutation reveals a conserved role for the carboxy-terminus in FMRP localization and function
Source: EMBO Mol Med. 2015 Feb 18;7(4):423–37. doi: 10.15252/emmm.201404576 (PMC4403044; doi:10.15252/emmm.201404576)

SUPPLEMENTAL DATA

Supplemental Figure Legends………………………………………………………………………… 2

Supplemental Figures 1-5…………………………………………………………………………...… 4

Supplemental Figure 1

G-insertion identified among previously reported variations in *FMR1*

(A) Summary of previously unreported intragenic variations in *FMR1* in 7 males. The subject of the study is indicated in bold.

(B) Guanine insertion in exon 15 [1457insG] of FMR1 identified in the subject of the study.

Supplemental Figure 2

Patient C-terminus encodes a functional nuclear localization signal (NLS).

(A) HEK293 cells transfected with vectors encoding mCherry-tagged wild type profilin *(mCherry-Profilin)* or mCherry-tagged profilin fused with the patient NLS sequence (*mCherry-Profilin^{+NLS}*). Wild type profilin is cytoplasmic, however, when fused to the NLS peptide, aggregates in nuclear inclusions.

Western blot analysis depicting expression levels of the different FMRP variants studied in the cell culture. A GFP-antibody was used on whole cell lysates of HEK293 cells transfected with the different GFP-FMR1 fusion constructs. Anti-actin was used for validation of protein load. Expression levels do not correlate with localization phenotypes. Scale bar represents 10μm.

Supplemental Figure 3

Sequence alignment of FMR/FXR protein family

(A) Amino acid alignment of human FMRP, FXR1P, FXR2P, mouse FMRP and fly FMRP. N-terminus is highly conserved while the C-terminus is more variable. The protein sequences (accession numbers # AAB28395.2, AAC50155.1, NP004851.2, NP032057.2, AAF54493.2) were aligned with the multiple sequence alignment (MUSCLE) function of MEGA6 (Tamura et al. 2013) using the default parameters and the alignment was corrected manually.

The black asterisk indicates the corresponding location of the patient mutation. The red asterisk shows the location of the C-terminus truncation engineered in the *dfmr1* variants (i.e. *dfmr1$^{\Delta Ct}$* and *dfmr1$^{\Delta Ct\ +NLS}$*).

Supplemental Figure 4

Protein levels of *dfmr1* variants tested

(A) A pan-neuronal driver (Elav-Gal4) was used to express the different *dfmr1* variants and protein expression levels were determined via western blot analysis of adult fly brain protein extracts. Anti-Gapdh was used for validation of protein load. The graph shows changes in expression level of the different Dfmrp isoforms relative to wild type. Expression levels do not correlate with axonal phenotypes. For example, the relative expression levels of Dfmrp^{wt+NLS} and Dfmrp $^{\Delta Ct+NLS}$ are comparable, yet unlike Dfmrp^{wt+NLS}, Dfmrp $^{\Delta Ct+NLS}$ fails to cause axonal collapse. Similarly, novel axonal misguidance phenotypes are only observed with Dfmrp $^{\Delta Ct+NLS}$ expression.

Supplemental Figure 5

Increasing Dfmrp expression in LNv neurons does not cause changes in axonal and subcellular localization phenotypes.

(A) Further increasing expression of wild type Dfmrp in LNv neurons does not alter axonal collapse phenotypes. As observed for one copy of UAS-dfmr1, two copies of UAS-dfmr1 cause strong axonal collapse. However, no misguidance phenotypes are observed (n=20). Scale bar represents 10µm.

(B) Independent of expression levels, Dfmrp is predominantly cytoplasmic in LNv neurons. Scale bar represents 5µm.

Supplemental Fig 1

A

Genomic Position	Type	Annotation
9896	A>G	intronic
10056	T>C	intronic
16812	G>A	Coding, silent
24421	C>T	intronic
32758	G>T	intronic
32825	delC	intronic
**33020**	**insG**	**coding, frameshift**

B

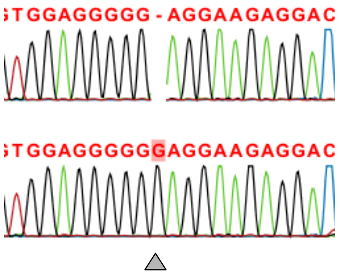

Supplemental Fig 2

A

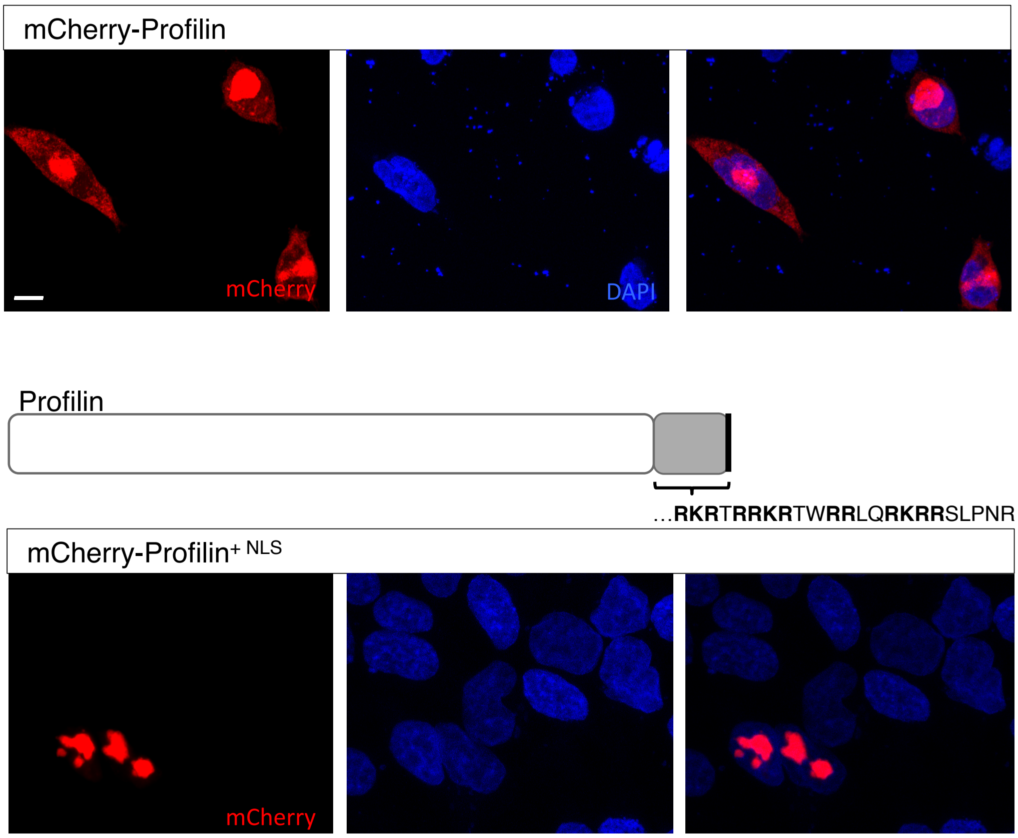

B

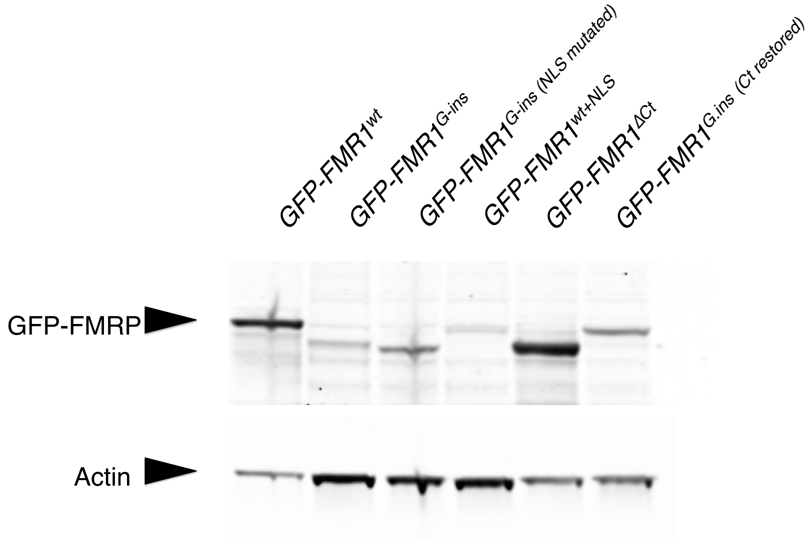

Supplemental Fig 2

A

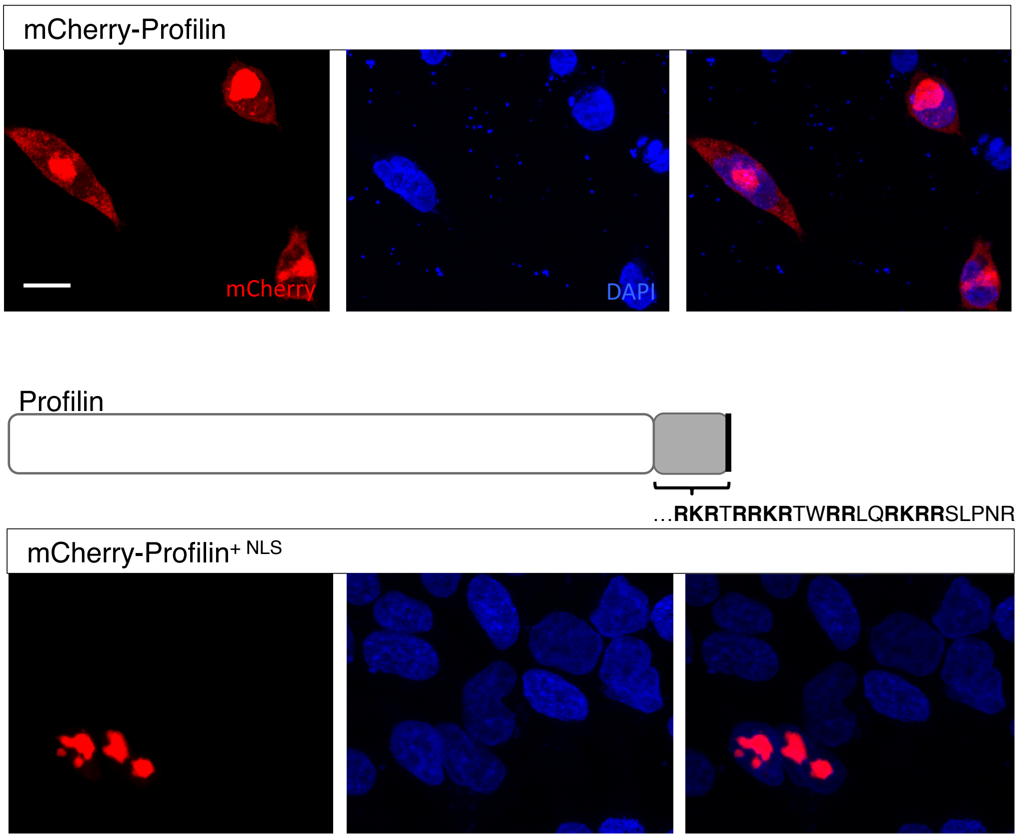

B

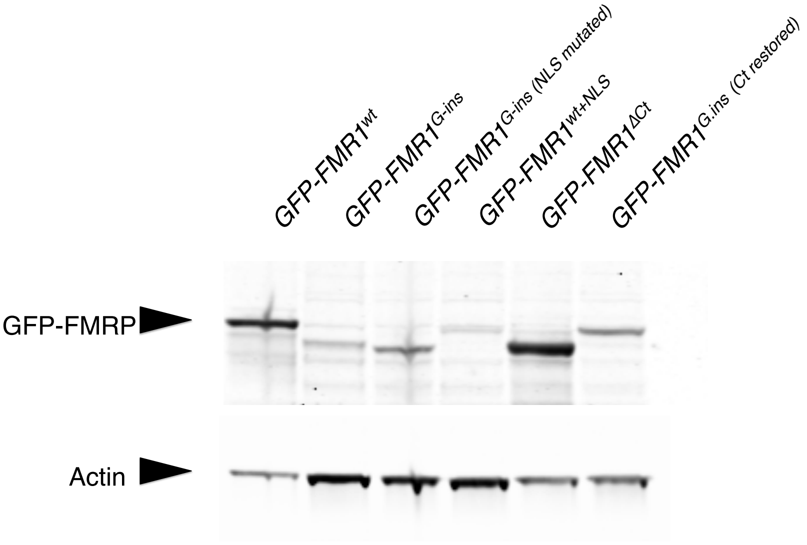

Supplemental Fig 3

A

```
        1
Hs-FMR1 ---------- MEELVVEVRG SNGAFYKAFV KDVHEDSITV AFENNWQPDR QIPFHDVRFP P--PVGYNKD
Hs-FXR1 ---------- MADVTVEVRG SNGAFYKGFI KDVHEDSLTV VFENNWQPER QVPFNEVRLP P--PPDIKKE
Hs-FXR2 MGGLASGGDV EPGLPVEVRG SNGAFYKGFV KDVHEDSVTI FFENNWQSER QIPFGDVRLP P--PADYNKE
Mm-FMR1 ---------- MEELVVEVRG SNGAFYKAFV KDVHEDSITV AFENNWQPER QIPFHDVRFP P--PVGYNKD
Dm-FMR1 ---------- MEDLLVEVRL DNGAYYKGQV TAVADDGIFV DVDG-VPESM KYPFVNVRLP PEETVEVAAP

        71
Hs-FMR1 INES-DEVEV YSRANEKEPC CWWLAKVRMI KGEFYVIEYA ACDATYNEIV TIERLRSVNP NKPATKDTFH
Hs-FXR1 ISEG-DEVEV YSRANDQEPC GWWLAKVRMM KGEFYVIEYA ACDATYNEIV TFERLRPVNQ NKTVKKNTFF
Hs-FXR2 ITEG-DEVEV YSRANEQEPC GWWLARVRMM KGDFYVIEYA ACDATYNEIV TLERLRPVNP NPLATKGSFF
Mm-FMR1 INES-DEVEV YSRANEKEPC CWWLAKVRMI KGEFYVIEYA ACDATYNEIV TIERLRSVNP NKPATKDTFH
Dm-FMR1 IFEEGMEVEV FTRTNDRETC GWWVGIIKMR KAEIYAVAYI GFETSYTEIC ELGRLRAKNS NPPITAKTFY

        141
Hs-FMR1 KIKLDVPEDL RQMCAKEAAH KDFKKAVGAF SVTYDPENYQ LVILSINEVT SKRAHMLIDM HFRSLRTKLS
Hs-FXR1 KCTVDVPEDL REACANENAH KDFKKAVGAC RIFYHPETTQ LMILSASEAT VKRVNILSDM HLRSIRTKLM
Hs-FXR2 KVTMAVPEDL REACSNENVH KEFKKALGAN CIFLNITNSE LFILSTTEAP VKRASLLGDM HFRSLRTKLL
Mm-FMR1 KIKLEVPEDL RQMCAKESAH KDFKKAVGAF SVTYDPENYQ LVILSINEVT SKRAHMLIDM HFRSLRTKLS
Dm-FMR1 QFTLPVPEEL REEAQKDGIH KEFQRTIDAG VCNYSRDLDA LIVISKFEHT QKRASMLKDM HFRNLSQKVM

        211
Hs-FMR1 LIMRNEEASK QLESSRQLAS R-FHEQFIVR EDLMGLAIGT HGANIQQARK VPGVTAIDLD EDTCTFHIYG
Hs-FXR1 LMSRNEEATK HLECTKQLAA A-FHEEFVVR EDLMGLAIGT HGSNIQQARK VPGVTAIELD EDTGTFRIYG
Hs-FXR2 LMSRNEEATK HLETSKQLAA A-FQEEFTVR EDLMGLAIGT HGANIQQARK VPGVTAIELG EETCTFRIYG
Mm-FMR1 LILRNEEASK QLESSRQLAS R-FHEQFIVR EDLMGLAIGT HGANIQQARK VPGVTAIDLD EDTCTFHIYG
Dm-FMR1 LLKRTEEAAR QLETTKLMSR GNYVEEFRVR DDLMGLAIGS HGSNIQAART VDGVTNIELE EKSCTFKISG

        281
Hs-FMR1 EDQDAVKKAR SFLEFAEDVI QVPRNLVGKV IGKNGKLIQE IVDKSGVVRV RIEA-----E NEKNVPQEEE
Hs-FXR1 ESADAVKKAR GFLEFVEDFI QVPRNLVGKV IGKNGKVIQE IVDKSGVVRV RIEG-----D NENKLPRED-
Hs-FXR2 ETPEACRQAR SYLEFSEDSV QVPRNLVGKV IGKNGKVIQE IVDKSGVVRV RVEG-----D NDKKNPREE-
Mm-FMR1 EDQDAVKKAR SFLEFAEDVI QVPRNLVGKV IGKNGKLIQE IVDKSGVVRV RIEA-----E NEKSVPQEEE
Dm-FMR1 ETEESVQRAR AMLEYAEEFF QVPRELVGKV IGKNGRIIQE IVDKSGVFRI KVSAIAGDDE QDQNIPREL-

        351
Hs-FMR1 IMPPNSLPSN NSRVGPNAPE EKKHLDIKEN STHFSQPNST KVQRVLVASS VVAGESQKPE LKAWQGMVPF
Hs-FXR1 ---------- ---------- ---------- ---------- ---------- ---------- -----GMVPF
Hs-FXR2 ---------- ---------- ---------- ---------- ---------- ---------- -----GMVPF
Mm-FMR1 IMPPSSLPSN NSRVGPNSSE EKKHLDTKEN -THFSQPNST KVQRVLVVSS IVAGGPQKPE PKAWQGMVPF
Dm-FMR1 ---------- ---------- ---------- ---------- ---------- ---------- -----AHVPF

        421
Hs-FMR1 VFVGTKDSIA NATVLLDYHL NYLKEVDQLR LERLQIDEQL RQIGASSRPP PNRTDK---- EKSYVTDDGQ
Hs-FXR1 VFVGTKESIG NVQVLLEYHI AYLKEVEQLR MERLQIDEQL RQIGS----- ---------- ----------
Hs-FXR2 IFVGTRENIS NAQALLEYHL SYLQEVEQLR LERLQIDEQL RQIGLGFRPP GSGRGSGGSD KAGYSTDESS
Mm-FMR1 VFVGTKDSIA NATVLLDYHL NYLKEVDQLR LERLQIDEQL RQIGASSRPP PNRTDK---- EKGYVTDDGQ
Dm-FMR1 VFIGTVESIA NAKVLLEYHL SHLKEVEQLR QEKMEIDQQL RAIQESSMGS TQSFPVTRRS ERGYSSDIES

        491
Hs-FMR1 GMGRGS---- -RPYRNRGHG RRGP--GYTS GTNSEASNAS ETESDH---- ---RDELSDW SLAPTEEERE
Hs-FXR1 ---------- -RSYSGRGRG RRGPNYTSGY GTNSELSNPS ETESER---- ---KDELSDW SLAGEDN-RD
Hs-FXR2 SSSLHATRTY GGSYGGRGRG RRTG--GPAY GPSSDVSTAS ETESEK---- ---REEP--- NRAGPGD-RD
Mm-FMR1 GMGRGS---- -RPYRNRGHG RRGP--GYTS GTNSEASNAS ETESDH---- ---RDELSDW SLAPTEEERE
Dm-FMR1 VRSMRGG--- GGGQRGRVRG RGGGGPGGGN GLNQRYHNNR RDEDDYNSRG DHQRDQQRGY ND--------

                          *   FMR/FXR RGG Box
        561
Hs-FMR1 SFLRRGDG-- -RRRGGGGRG QG---GRGRG GG-------- --FKGNDD-- HSRTDNRP-- ----------
Hs-FXR1 SRHQ-RDS-- -RRRP-GGRG RSVSGGRGRG GPRGGKSSIS SVLKDPDSNP YSLLDNTESD QTADTDASES
Hs-FXR2 PPTRGEES-- -RRRPTGGRG RGPPPAP-RP TSRYNSSSIS SVLKDPDSNP YSLLDTSEPE PPVDSEPGEP
Mm-FMR1 SFLRRGDG-- -RRRGGGGRG QG---GRGRG GG-------- --FKGNDD-- HSRTDNRP-- ----------
Dm-FMR1 ---RGGGDNT GSYRGGGGGA GGPGNNR-RG GINRRPPRND QQNGRD---- -YQHHNHTTE EVRETREMSS

                          *
        631
Hs-FMR1 ---------- ----RNPREA KGRTTDGSLQ IRVDCNNER- SVHTKTLQNT -----SSEGS RLRTGKDRN-
Hs-FXR1 HHSTN----- ----RRRRSR RRRTDEDAVL MDGMTESDTA SVNENGLVTV -----ADYIS RAESQSRQR-
Hs-FXR2 PPASA----- ----RRRRSR RRRTDEDRTV MDGGLESDGP NMTENGLEDE -----SRPQR RNRSRRRRNR
Mm-FMR1 ---------- ----RNPREA KGRTADG--- ---------- -----SLQSA -----SSEGS RLRTGKDRN-
Dm-FMR1 VERADSNSSY EGSSRRRRRQ KNNNGPSNTN GAVANNNNKP QSAQQPQQQQ PPAPGNKAAL NAGDASKQNS

        701
Hs-FMR1 ---------- ---------- ---------- -----QKKEK PDSVDG---- ---------- ----------
Hs-FXR1 -NLPRETLAK NKK------- -EMAKDVIEE HGPSEKAING PTSASG---D DISKLQRTPG EEKINTLKEE
Hs-FXR2 GNRTDGSISG DRQPVTVADY ISRAESQSRQ RPPLERTKPS EDSLSGQKGD SVSKLPKGPS E---NGELSA
Mm-FMR1 ---------- ---------- ---------- -----QKKEK PDSVDG---- ---------- ----------
Dm-FMR1 GNANAAGGAS KPKDASRNGD KQQAGTQQQQ PSQVQQQQAA QQQQPKPR-R NKNRSNNHTD QPSGQQQLAE

        771
Hs-FMR1 ---QQPLVNG VP
Hs-FXR1 NTQEAAVLNG VS
Hs-FXR2 PLELGSMVNG VS
Mm-FMR1 ---LQPLVNG VP
Dm-FMR1 NVKKEGLVNG TS
```

Supplemental Fig 4

A

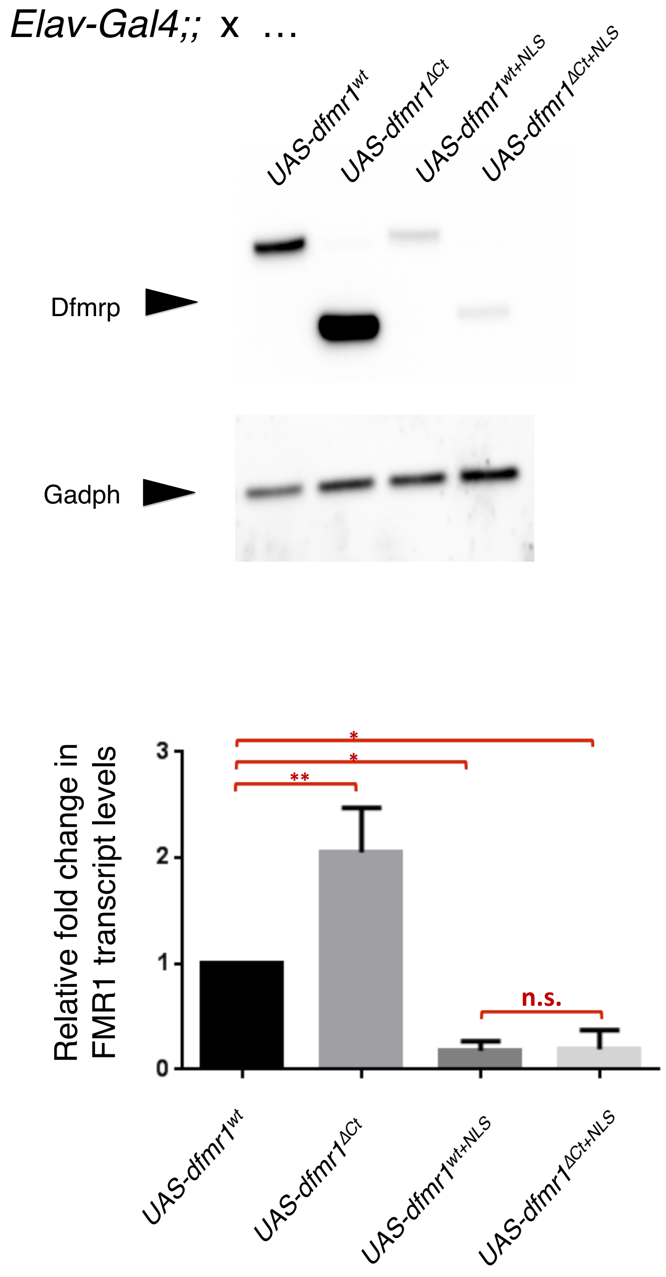

Supplemental Fig 5

A

Pdf-Gal4, UAS-CD8-GFP x …

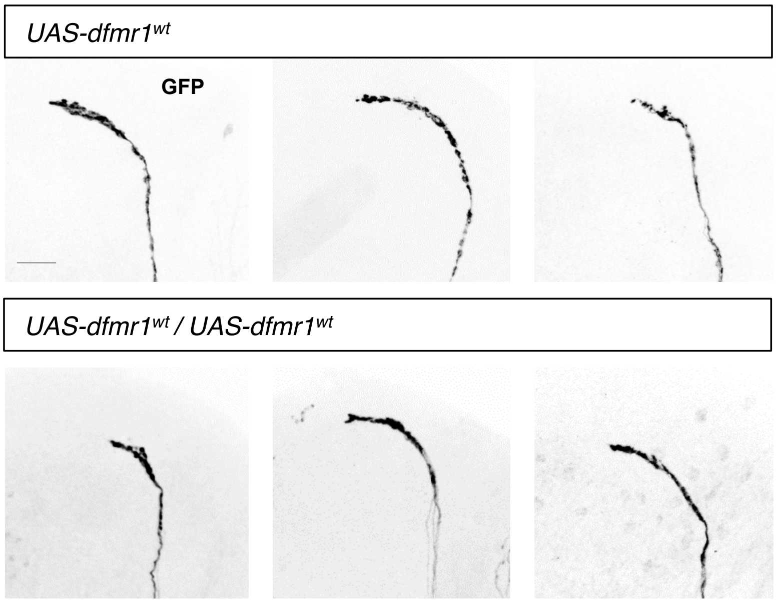

B

Pdf-Gal4, UAS-CD8-GFP x …

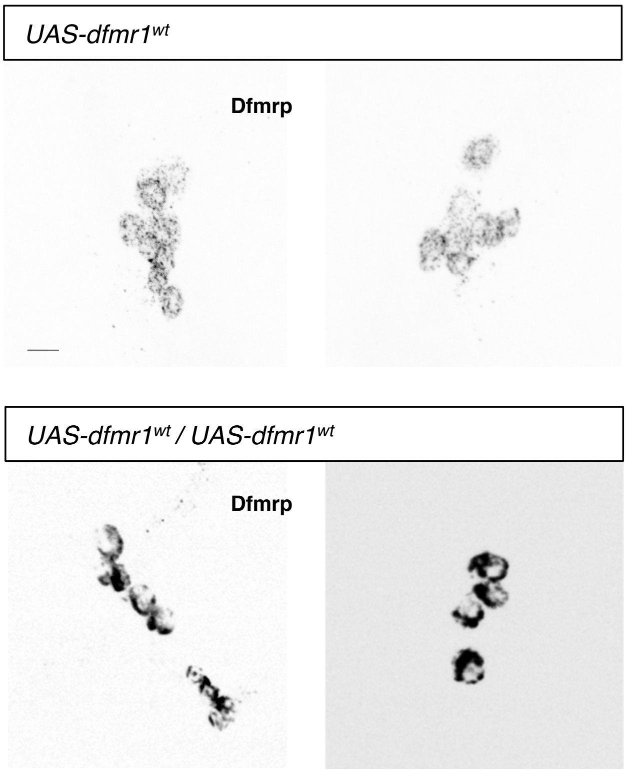

Supplement: Supplementary file 1 — Supplementary Figures S1–S5 [file emmm0007-0423-sd1.pdf]
